# Supplementary material for: A Mediterranean Diet Is Positively Associated with Bone and Muscle Health in a Non-Mediterranean Region in 25,450 Men and Women from EPIC-Norfolk
Source: Nutrients. 2020 Apr 21;12(4):1154. doi: 10.3390/nu12041154 (PMC7231007; doi:10.3390/nu12041154)

Table S1: Characteristics of participants' diets by quintile of Alternative Mediterranean Diet Score in 25,450 men and women aged 39-79 y

	Q1	Q2	Q3	Q4	Q5	P=
	n=5161	n=4991	n=5557	n=4740	n=5001	
aMED score, range	0-2	3	4	5	6-9	-
**Components of aMED score**						
Vegetables, g/d	76.6 (74.7,78.5)	97.2 (95.3,99.2)	109 (107,111)	123 (121,125)	143 (141,145)	<0.01
Legumes, g/d	7.31 (6.63,7.99)	11.7 (11.0,12.4)	14.3 (13.7,15.0)	16.3 (15.6,17.0)	20.2 (19.5,20.9)	<0.01
Fruit, g/d	86.1 (82.7,89.4)	128 (125,131)	161 (158,164)	193 (190,197)	235 (232,239)	<0.01
Fish, g/d	19.4 (18.4,20.4)	31.6 (30.7,32.6)	39.2 (38.3,40.1)	45.6 (44.7,46.6)	56.1 (55.2,57.1)	<0.01
Nuts and seeds, g/d	0.51 (0.31,0.71)	1.40 (1.20,1.60)	2.00 (1.81,2.19)	3.22 (3.02,3.43)	5.22 (5.01,5.42)	<0.01
Wholegrains, g/d	8.35 (7.24,9.45)	17.2 (16.1,18.3)	26.6 (25.5,27.6)	36.4 (35.3,37.6)	46.1 (45.0,47.2)	<0.01
Red and processed meat, g/d	79.4 (78.3,80.5)	68.4 (67.4,69.5)	61.6 (60.6,62.6)	54.6 (53.5,55.6)	44.6 (43.5,45.6)	<0.01
Dairy, g/d	281 (276,285)	277 (272,281)	275 (271,279)	268 (264,272)	272 (267,276)	<0.01
Ratio MUFA: SFA	0.84 (0.84,0.85)	0.91 (0.91,0.92)	0.94 (0.94,0.95)	0.99 (0.98,0.99)	1.04 (1.04,1.05)	<0.01
Alcohol, g/d	11.3 (10.8,11.7)	12.0 (11.5,12.5)	12.2 (11.8,12.6)	12.2 (11.7,12.6)	12.0 (11.5,12.4)	0.05
**Dietary intake**						
Energy, kcal/d	1934 (1930,1938)	1937 (1934,1941)	1942 (1938,1945)	1945 (1941,1949)	1947 (1943,1951)	<0.01
Vitamin C, mg/d	67.3 (65.9,68.6)	78.8 (77.5,80.1)	87.0 (85.8,88.3)	97.2 (95.8,98.6)	109 (108,111)	<0.01
Vitamin D, mcg/d	2.69 (2.63,2.76)	3.06 (2.99,3.12)	3.31 (3.25,3.37)	3.56 (3.50,3.63)	3.88 (3.82,3.95)	<0.01
Magnesium, mg/d	255 (253,257)	274 (272,276)	290 (289,292)	308 (306,310)	334 (332,335)	<0.01
Calcium, mg/d	827 (821,833)	828 (822,834)	834 (828,839)	839 (832,845)	850 (844,856)	<0.01
Potassium, mg/d	2888 (2872,2903)	3064 (3049,3079)	3199 (3185,3214)	3336 (3321,3352)	3543 (3528,3559)	<0.01
Protein, %E	15.0 (14.9,15.0)	15.2 (15.1,15.3)	15.4 (15.3,15.5)	15.6 (15.5,15.6)	15.8 (15.7,15.9)	<0.01
Saturated fat, %E	14.6 (14.6,14.7)	13.5 (13.4,13.6)	12.8 (12.7,12.9)	12.2 (12.1,12.2)	11.3 (11.3,11.4)	<0.01
Monounsaturated fat, %E	12.1 (12.0,12.2)	12.0 (11.9,12.0)	11.8 (11.8,11.9)	11.7 (11.6,11.7)	11.5 (11.5,11.6)	<0.01
Polyunsaturated fat, %E	6.10 (6.05,6.15)	6.40 (6.35,6.45)	6.57 (6.52,6.61)	6.68 (6.63,6.73)	6.91 (6.86,6.95)	<0.01
n-3 PUFA, g/d	1.28 (1.27,1.30)	1.44 (1.43,1.46)	1.55 (1.54,1.57)	1.66 (1.64,1.67)	1.83 (1.81,1.84)	<0.01
α-Linolenic acid , g/d	1.05 (1.04,1.06)	1.13 (1.12,1.14)	1.18 (1.17,1.19)	1.22 (1.21,1.23)	1.27 (1.26,1.28)	<0.01
Eicosapentaenoic acid, g/d	0.06 (0.05,0.06)	0.08 (0.08,0.09)	0.10 (0.10,0.10)	0.12 (0.12,0.13)	0.16 (0.15,0.16)	<0.01
Docosahexaenoic acid, g/d	0.07 (0.06,0.08)	0.11 (0.11,0.12)	0.14 (0.14,0.15)	0.18 (0.17,0.18)	0.24 (0.23,0.24)	<0.01

Values are adjusted means (95% CI), n=25450. Means were adjusted for sex, age, BMI, smoking, physical activity, number of days food diary completed and under-reporting of energy intake. aMED= Alternative Mediterranean Diet Score

Table S2: Characteristics of participants' diets by quintile of Mediterranean Diet Score in 25,450 men and women aged 39-79 y

	Q1	Q2	Q3	Q4	Q5	P=
	n=3304	n=4779	n=5918	n=5681	n=5768	
MDS score, range	0-2	3	4	5	6-9	-
**Components of MDS score**						
Vegetables, g/d	78.0 (75.6,80.4)	92.5 (90.6,94.5)	104 (102,106)	118 (116,120)	139 (137,141)	<0.01
Legumes, g/d	6.27 (5.43,7.10)	10.2 (9.54,10.9)	12.9 (12.3,13.5)	15.6 (15.0,16.2)	20.7 (20.1,21.4)	<0.01
Fruit and nuts, g/d	94.0 (89.8,98.3)	126 (123,130)	154 (151,157)	184 (180,187)	219 (216,222)	<0.01
Fish, g/d	18.9 (17.7,20.1)	27.1 (26.1,28.1)	36.1 (35.3,37.0)	44.1 (43.2,45.0)	54.9 (54.0,55.8)	<0.01
Cereals, g/d	208 (205,211)	226 (223,228)	236 (234,239)	249 (247,251)	268 (266,270)	<0.01
Meat and eggs, g/d	160 (158,163)	141 (139,143)	129 (128,131)	115 (114,117)	101 (99.6,103)	<0.01
Dairy, g/d	353 (347,358)	304 (300,308)	277 (274,281)	254 (250,258)	223 (219,227)	<0.01
Ratio MUFA: SFA	0.83 (0.83,0.84)	0.89 (0.88,0.89)	0.93 (0.93,0.94)	0.98 (0.97,0.98)	1.03 (1.03,1.04)	<0.01
Alcohol, g/d	10.7 (10.1,11.2)	11.1 (10.6,11.5)	12.1 (11.7,12.5)	12.0 (11.6,12.4)	13.1 (12.7,13.5)	<0.01
**Dietary intake**						
Energy, kcal/d	1932 (1928,1937)	1937 (1933,1941)	1941 (1937,1944)	1941 (1937,1945)	1950 (1946,1954)	<0.01
Vitamin C, mg/d	70.1 (68.4,71.7)	77.8 (76.4,79.2)	85.2 (83.9,86.4)	92.6 (91.3,93.8)	104 (103,105)	<0.01
Vitamin D, mcg/d	2.79 (2.71,2.87)	2.99 (2.93,3.06)	3.23 (3.17,3.28)	3.40 (3.34,3.46)	3.80 (3.74,3.86)	<0.01
Magnesium, mg/d	269 (267,272)	277 (275,279)	288 (286,290)	297 (295,299)	316 (314,318)	<0.01
Calcium, mg/d	886 (879,893)	851 (844,857)	836 (830,841)	822 (816,828)	807 (801,813)	<0.01
Potassium, mg/d	3018 (2998,3038)	3083 (3066,3099)	3168 (3153,3183)	3249 (3234,3264)	3398 (3383,3413)	<0.01
Protein, %E	15.5 (15.4,15.6)	15.3 (15.3,15.4)	15.4 (15.3,15.4)	15.3 (15.3,15.4)	15.4 (15.3,15.5)	0.19
Saturated fat, %E	14.9 (14.8,15.0)	13.9 (13.8,13.9)	13.1 (13.0,13.1)	12.3 (12.3,12.4)	11.3 (11.3,11.4)	<0.01
Monounsaturated fat, %E	12.2 (12.1,12.2)	12.0 (12.0,12.1)	11.9 (11.8,11.9)	11.8 (11.7,11.8)	11.4 (11.4,11.5)	<0.01
Polyunsaturated fat, %E	5.78 (5.72,5.84)	6.23 (6.18,6.28)	6.49 (6.45,6.54)	6.75 (6.71,6.80)	7.02 (6.97,7.06)	<0.01
n-3 PUFA, g/d	1.30 (1.28,1.32)	1.41 (1.39,1.42)	1.51 (1.50,1.52)	1.62 (1.60,1.63)	1.78 (1.77,1.80)	<0.01
α-Linolenic acid , g/d	1.06 (1.05,1.07)	1.12 (1.11,1.13)	1.15 (1.15,1.16)	1.21 (1.20,1.22)	1.26 (1.25,1.26)	<0.01
Eicosapentaenoic acid, g/d	0.06 (0.05,0.06)	0.07 (0.07,0.08)	0.10 (0.09,0.10)	0.11 (0.11,0.12)	0.15 (0.15,0.16)	<0.01
Docosahexaenoic acid, g/d	0.07 (0.07,0.08)	0.10 (0.10,0.11)	0.14 (0.13,0.14)	0.16 (0.16,0.17)	0.22 (0.22,0.23)	<0.01

Values are adjusted means (95% CI), n=25450. Means were adjusted for sex, age, BMI, smoking, physical activity, number of days food diary completed and under-reporting of energy intake. MDS= Mediterranean Diet Score.

Table S3: Fat free mass (kg) by quintile of Alternative Mediterranean Diet Score and Mediterranean Diet Score in 14,720 men and women aged 42-82 y, stratified by sex[1]

Sex	Quintile	aMED		MDS	
		n=	Mean (95% CI)	n=	Mean (95% CI)
**All**	Q1	2711	49.3 (49.1,49.5)	1852	49.1 (48.9,49.4)
	Q2	2848	49.6 (49.4,49.8)	2668	49.5 (49.3,49.7)
	Q3	3212	50.0 (49.8,50.1)	3446	49.9 (49.7,50.1)
	Q4	2914	50.0 (49.8,50.1)	3314	49.9 (49.7,50.1)
	Q5	3035	50.3 (50.1,50.4)	3440	50.4 (50.2,50.5)
	Q5-Q1		0.92 (0.65, 1.19)		1.21 (0.92, 1.51)
	P=		<0.01		<0.01
**Men**	Q1	1155	61.3 (61.0,61.6)	749	61.0 (60.6,61.3)
	Q2	1208	61.5 (61.3,61.8)	1123	61.4 (61.1,61.7)
	Q3	1423	61.7 (61.5,62.0)	1497	61.7 (61.5,62.0)
	Q4	1299	61.9 (61.6,62.2)	1486	61.8 (61.5,62.1)
	Q5	1361	62.1 (61.8,62.4)	1591	62.3 (62.0,62.5)
	Q5-Q1		0.79 (0.37, 1.21)		1.31 (0.85, 1.77)
	P=		<0.01		<0.01
**Women**	Q1	1556	40.1 (39.8,40.3)	1103	40.0 (39.7,40.3)
	Q2	1640	40.3 (40.1,40.6)	1545	40.2 (40.0,40.4)
	Q3	1789	40.8 (40.5,41.0)	1949	40.7 (40.5,40.9)
	Q4	1615	40.7 (40.5,40.9)	1828	40.6 (40.4,40.9)
	Q5	1674	41.0 (40.8,41.2)	1849	41.0 (40.8,41.2)
	Q5-Q1		0.95 (0.62, 1.29)		0.96 (0.60, 1.32)
	P=		<0.01		<0.01

[1]Values are adjusted means (95% CI), n=14815. Means were adjusted for sex, age, BMI, smoking, physical activity, family history of osteoporosis, calcium intakes, supplement use (vitamin D or calcium), medication use (corticosteroids, aspirin or hormone replacement therapy), menopausal status, days of dietary intake data and the ratio of energy intake to estimated energy requirements. P values are for trends calculated using ANCOVA. aMED= Alternative Mediterranean Diet Score; MDS = Mediterranean Diet Score.

Table S4: Measures of broadband ultrasound attenuation, velocity of sound and fat free mass by quintile of alternative Mediterranean Diet Score in 14,815 men and women aged 42-82 y, stratified by sex[1]

Sex	Quintile	BUA (dB/MHz)		VOS (m/s)		FFM^{BMI} (kg/[kg/m²])	
		n=	Mean (95% CI)	n=	Mean (95% CI)	n=	Mean (95% CI)
**All**	Q1	2725	79.3 (78.6,80.0)	2725	1631 (1630,1633)	2711	1.85 (1.83,1.87)
	Q2	2865	79.0 (78.3,79.7)	2865	1632 (1630,1633)	2848	1.87 (1.85,1.89)
	Q3	3240	80.3 (79.7,81.0)	3240	1635 (1633,1636)	3212	1.90 (1.88,1.92)
	Q4	2928	80.2 (79.5,80.9)	2928	1634 (1633,1636)	2914	1.91 (1.89,1.93)
	Q5	3057	80.7 (80.0,81.4)	3057	1636 (1634,1637)	3035	1.94 (1.92,1.96)
	Q5-Q1		1.42 (0.43, 2.41)		4.41 (2.27, 6.55)		0.09 (0.07, 0.11)
	P=		<0.01		<0.01		<0.01
**Men**	Q1	1157	89.8 (88.8,90.8)	1157	1643 (1641,1645)	1155	2.29 (2.28,2.31)
	Q2	1213	89.5 (88.5,90.5)	1213	1643 (1641,1646)	1208	2.30 (2.29,2.32)
	Q3	1442	90.0 (89.1,90.9)	1442	1645 (1643,1648)	1423	2.31 (2.30,2.33)
	Q4	1303	90.3 (89.4,91.3)	1303	1646 (1643,1648)	1299	2.33 (2.32,2.34)
	Q5	1375	90.6 (89.7,91.6)	1375	1648 (1646,1650)	1361	2.35 (2.34,2.36)
	Q5-Q1		0.86 (-0.51, 2.23)		4.99 (1.87, 8.11)		0.06 (0.04, 0.08)
	P=		0.10		<0.01		<0.01
**Women**	Q1	1568	71.6 (70.7,72.4)	1568	1623 (1621,1625)	1556	1.52 (1.51,1.54)
	Q2	1652	71.3 (70.5,72.1)	1652	1623 (1621,1625)	1640	1.55 (1.54,1.56)
	Q3	1798	72.6 (71.8,73.3)	1798	1626 (1624,1628)	1789	1.57 (1.56,1.59)
	Q4	1625	72.2 (71.4,73.0)	1625	1625 (1623,1627)	1615	1.57 (1.56,1.59)
	Q5	1682	72.6 (71.8,73.4)	1682	1626 (1624,1627)	1674	1.61 (1.59,1.62)
	Q5-Q1		1.05 (-0.08, 2.18)		2.99 (0.23, 5.76)		0.08 (0.06, 0.10)
	P=		0.02		0.01		<0.01

[1]Values are unadjusted means (95% CI), n=14815. P values are for trends calculated using ANCOVA. BUA=Broadband ultrasound attenuation; VOS = velocity of sound; FFM^{BMI} =Fat free mass adjusted for BMI.

Table S5: Measures of broadband ultrasound attenuation, velocity of sound and fat free mass by quintile of Mediterranean Diet Score in 14,815 men and women aged 42-82 y, stratified by sex[1]

Sex	Quintile	BUA (dB/MHz)		VOS (m/s)		FFM^{BMI} (kg/[kg/m^2])	
		n=	Mean (95% CI)	n=	Mean (95% CI)	n=	Mean (95% CI)
**All**	Q1	1872	78.5 (77.6,79.3)	1872	1630 (1628,1632)	1852	1.84 (1.82,1.86)
	Q2	2669	79.4 (78.7,80.2)	2669	1633 (1631,1635)	2668	1.87 (1.85,1.89)
	Q3	3468	79.7 (79.0,80.3)	3468	1633 (1632,1634)	3446	1.89 (1.88,1.91)
	Q4	3350	80.3 (79.6,80.9)	3350	1635 (1633,1636)	3314	1.91 (1.89,1.92)
	Q5	3456	81.1 (80.4,81.7)	3456	1636 (1635,1637)	3440	1.94 (1.92,1.96)
	Q5-Q1		2.61 (1.54, 3.69)		6.41 (4.08, 8.73)		0.10 (0.07, 0.13)
	P=		<0.01		<0.01		<0.01
**Men**	Q1	754	88.9 (87.6,90.1)	754	1642 (1639,1645)	749	2.29 (2.28,2.31)
	Q2	1122	90.0 (89.0,91.1)	1122	1645 (1643,1647)	1123	2.30 (2.29,2.32)
	Q3	1511	90.3 (89.4,91.2)	1511	1646 (1644,1648)	1497	2.32 (2.30,2.33)
	Q4	1504	89.6 (88.7,90.4)	1504	1644 (1642,1646)	1486	2.32 (2.31,2.33)
	Q5	1599	90.9 (90.0,91.7)	1599	1648 (1646,1650)	1591	2.34 (2.33,2.35)
	Q5-Q1		2.03 (0.51, 3.55)		5.87 (2.41, 9.32)		0.04 (0.02, 0.07)
	P=		0.05		0.01		<0.01
**Women**	Q1	1118	71.5 (70.5,72.4)	1118	1621 (1619,1624)	1103	1.53 (1.51,1.55)
	Q2	1547	71.8 (70.9,72.6)	1547	1624 (1622,1626)	1545	1.55 (1.54,1.56)
	Q3	1957	71.5 (70.7,72.2)	1957	1623 (1621,1625)	1949	1.56 (1.55,1.58)
	Q4	1846	72.7 (72.0,73.5)	1846	1627 (1625,1629)	1828	1.57 (1.56,1.58)
	Q5	1857	72.6 (71.9,73.4)	1857	1626 (1624,1628)	1849	1.60 (1.58,1.61)
	Q5-Q1		1.18 (-0.05, 2.40)		4.54 (1.56, 7.53)		0.07 (0.05, 0.09)
	P=		0.01		<0.01		<0.01

[1]Values are unadjusted means (95% CI), n=14815. P values are for trends calculated using ANCOVA. BUA=Broadband ultrasound attenuation; VOS = velocity of sound; FFM^{BMI} =Fat free mass adjusted for BMI.

Figure S1: Total fracture risk after a mean follow up of 17.6 years by quintile of alternative Mediterranean Diet Score and Mediterranean Diet Score in 8351 post-menopusal women aged 40-78 y.

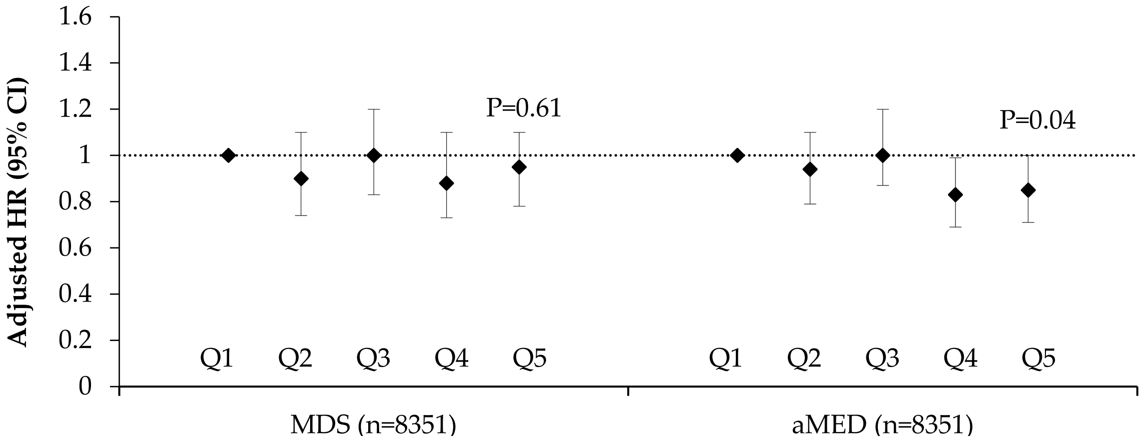

Values are adjusted hazard ratios (95% CI), n=25,450. Ratios were adjusted for age, BMI, smoking, physical activity, family history of osteoporosis, calcium intakes, supplement use (vitamin D or calcium), medication use (corticosteroids, aspirin or hormone replacement therapy), days of dietary intake data and the ratio of energy intake to estimated energy requirements. P values are for trends calculated using Cox proportional hazards model. Participant numbers (cases) per quintile were as follows; MDS: Q1= 1158 (188), Q2= 1679 (245), Q3= 1968 (327), Q4= 1833 (268), Q5= 1713 (260); aMED: Q1= 1769 (287), Q2= 1711 (262), Q3= 1816 (307), Q4= 1534 (215), Q5= 1521 (217). aMED= alternative Mediterranean Diet Score, MDS= Mediterranean Diet Score.

Figure S2: Total fracture risk after a mean follow up of 17.6 years by quintile of aMED and MDS in 25,450 men and women aged 39-79 y. Values are unadjusted hazard ratios (95% CI), n=25,450. P values are for trends calculated using Cox proportional hazards model. Participant numbers (cases) per quintile were as follows; (a) aMED all participants: Q1= 5161 (481), Q2= 4991 (430), Q3= 5557 (515), Q4= 4740 (396), Q5= 5001 (373) (b) MDS all participants: Q1= 3304 (317), Q2= 4779 (416), Q3= 5918 (528), Q4= 5681 (494), Q5= 5768 (440). aMED= alternative Mediterranean Diet Score, MDS= Mediterranean Diet Score.

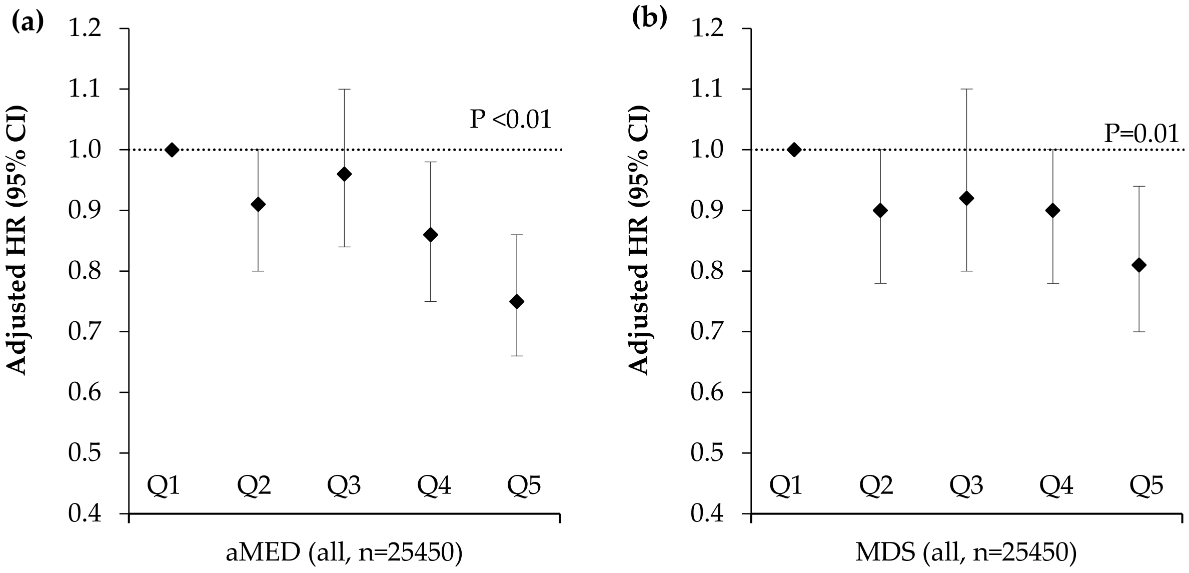

Supplement: Supplementary file 1 [file nutrients-12-01154-s001.pdf]
